# Uphill battle: Innovation of thiopurine therapy in global inflammatory bowel disease care

**DOI:** 10.1007/s12664-024-01529-x

**Published:** 2024-02-21

**Authors:** Ahmed B. Bayoumy, Chris J. J. Mulder, Azhar R. Ansari, Murray L. Barclay, Tim Florin, Marianne Kiszka-Kanowitz, Luc Derijks, Vishal Sharma, Nanne K. H. de Boer

**Affiliations:** 1 Department of Internal Medicine, Amsterdam University Medical Centers, Amsterdam, The Netherlands; 2 Department of Gastroenterology and Hepatology, AGEM Research Institute, Amsterdam University Medical Center, Vrije Universiteit Amsterdam, De Boelelaan 1117, 1081 HV Amsterdam, The Netherlands; 3 Department of Gastroenterology and Hepatology, London Bridge Hospital, London, UK; 4 Department of Gastroenterology, Christchurch Hospital, Christchurch, Waitaha - Canterbury New Zealand; 5 Department of Clinical Pharmacology, Christchurch Hospital, Christchurch, Waitaha - Canterbury New Zealand; 6 Mater Research, University of Queensland, Translational Research Institute, South Brisbane, Australia; 7 Copenhagen Center for Inflammatory Bowel Disease in Children, Adolescents and Adults, Copenhagen University Hospital - Amager and Hvidovre Hospital, Hvidovre, Denmark; 8 Department of Clinical Pharmacy, Máxima Medical Center, Veldhoven, The Netherlands; 9 Department of Clinical Pharmacy and Toxicology, Maastricht University Medical Center, Maastricht, The Netherlands; 10 Department of Gastroenterology, Postgraduate Institute of Medical Education and Research, Chandigarh 160 012, India

**Keywords:** Allopurinol, Azathioprine, Crohn’s disease, Inflammatory bowel disease, Mercaptopurine, Thioguanine, Thiopurines, Ulcerative colitis

## Abstract

Inflammatory bowel disease (IBD) is a chronic inflammatory disorder of the gastrointestinal tract that encompasses two major conditions: Crohn’s disease (CD) and ulcerative colitis (UC). Historically, IBD has been primarily reported in western countries, but over the past decades, its prevalence is rapidly increasing, especially in lower and middle-income countries (LMICs) such as India and China and also in Sub-Saharan Africa. The prevalence of IBD in LMICs has been the subject of growing concern due to the impact of access to public healthcare and the burden it places on healthcare resources. The classical thiopurines face significant challenges due to cessation of therapy in approximately half of patients within one year due to side effects or ineffectiveness. In this article, we highlight innovating thiopurine treatment for IBD patients in downregulating side effects and improving efficacy.

## Introduction

Inflammatory bowel disease (IBD) is a chronic inflammatory disorder of the gastrointestinal tract that encompasses two major conditions: Crohn’s disease (CD) and ulcerative colitis (UC) [1, 2]. Historically, IBD has been primarily reported in western countries, but over the past decades, its prevalence is rapidly increasing, especially in lower and middle-income countries (LMICs) such as India and China and also in Sub-Saharan Africa [3]. Behcet’s disease is well recognized along the Silk Road as part of IBD, but nearly mentioned elsewhere. This rising trend has posed challenges for access to care of underprivileged patients in these regions, necessitating a better understanding of the factors contributing to the increased prevalence of IBD [4]. Furthermore, the global IBD visualization of epidemiology studies in the 21st Century (GIVES-21) has been developed to investigate the epidemiology of IBD and explores new research questions on the association between environmental, dietary, genetic factors and IBD development in newly industrialized countries [5]. The prevalence of IBD in patients from LMICs has been the subject of growing concern due to the impact of access to public healthcare and the burden it places on healthcare resources [4]. While the rise in IBD cases is multi-factorial and complex, several key factors have been recognized. One significant factor is the westernization of lifestyle and dietary habits, changing the gut microbiota and increasing exposure to environmental risk factors [6]. As access to healthcare and diagnostics improves, more individuals are being diagnosed, leading to a recognized increase in IBD prevalence rates [7]. The impact of IBD goes beyond the burden on individuals and healthcare systems. It also affects work productivity, quality of life and economic costs associated with its management [6]. The challenges are further exacerbated by limited resources, inadequate access to specialized care and the limited availability of (expensive) medications [8]. Recently, Banerjee et al. [9] published a cross-sectional study of IBD demographics, disease phenotype and treatment across 38 centers in 15 countries of South Asia, South-East Asia and Middle East within the IBD-Emerging Nations’ Consortium (IBD-ENC). Their study consisted of a cohort of over 10,000 patients from 15 countries in the same geographic belt, where epidemiological data on IBD are underreported. They found that in the IBD-ENC, the UC is twice as common as CD, familial disease is uncommon and the rates of surgery are low. The use of biologics was correlated to the capita gross national income per capita, which also suggests that the most financially disadvantaged patients do not have access to these treatments. Hence, there is a need for medications that are effective, available and affordable. Globally, there are approximately 4.9 million cases of IBD worldwide, wherein thiopurines are the key therapy in a majority of IBD patients, not only because of their affordability [10–12]. Thiopurines are immunosuppressive medications used in various autoimmune and inflammatory conditions, including IBD [13]. Thiopurines were developed by Gertrude B. Elion and George H. Hitchings in the 1950s. They initially investigated thiopurines for their potential as an anticancer agent, but later recognized their immunosuppressive properties [14]. Thiopurines have demonstrated their efficacy in managing IBD and are affordable [15, 16]. The classical thiopurines, azathioprine and 6-MP, unfortunately have a relatively narrow therapeutic window and have adverse effects such as gastric intolerance, flu-like syndrome, pancreatitis, hepatotoxicity, skin rash, myelotoxicity and development of infections secondary to neutropenia [17]. The adverse effects are partly related to genetic variations in the metabolism of thiopurines. Because of this, the classical thiopurines face significant challenges such as monitoring of therapy and, if available, using therapeutic drug monitoring (TDM). Furthermore, genetic polymorphism testing for thiopurine S-methyltransferase (TPMT) and nudix hydrolase 15 (NUDT15) may also benefit patients, if testing for these polymorphisms is available and afforable [18]. The main reasons to monitor therapy include risk of hepatotoxicity and myelotoxicity. In this article, we highlight how to innovate strategies that are mandatory for IBD patients in downregulating side effects and improving efficacy.

## Azathioprine or mercaptopurine

Maintaining remission in IBD is crucial for improving patients’ quality of life and preventing disease progression. Azathioprine (AZA) (1.0–3.0 mg/kg) and mercaptopurine (MP) (1.0–1.5 mg/kg) have demonstrated, since the early 1980s, efficacy in reducing disease activity and promoting long-term remission in various Cochrane reviews. Based on evidence, the classical thiopurines are used for the maintenance of (surgically induced) remission in participants with CD [19]. AZA is effective for the maintenance of remission in ulcerative colitis. Furthermore, AZA or MP may be effective as maintenance therapy for patients who have failed or cannot tolerate mesalazine and for patients who require repeated courses of steroids [16]. The mesalazine treatments, in contrast to thiopurines, are not always affordable for underprivileged populations in countries such as India (Table 3) [20]. AZA seems more effective for the maintenance of remission in Crohn’s disease [15, 21]. A majority of drug trials are primarily performed with prescriptions under patent in high-income countries (HIC). Studies from rapidly rising countries have also demonstrated positive benefits from generic thiopurine therapies in IBD. Sood et al. [22] reported recently the effectiveness and safety of AZA in Indian UC patients. They reported that in patients who were steroid-refractory, steroid-dependent or had frequent relapse, the mean numbers of relapses prior to and post initiation of AZA therapy were 3.3 (± 0.8) and 0.9 (± 0.3), respectively (*p* < 0.01). They only recognized 16.2% (18/111) of patients who required discontinuation of AZA, lower than the usual rates reported in western countries. Ranjan et al. [23] reported results of 988 IBD patients (UC = 73%) on thiopurines (MP or AZA). They reported median efficacy rates of 79% and 72% at five years and 68% and 61% at 10 years in UC and CD patients, respectively, after classical dosing of AZA or MP. Yewale et al. [24] publish long-term real-world data from 320 Indian IBD patients treated with AZA. Approximately 20.6% of patients experienced side effects, which included myelotoxicity (7.2%) and gastrointestinal intolerance (5.6%). They also reported that 38.1% of patients had relapses requiring corticosteroid therapy and 16.2% had more than one relapse after AZA use. AZA was continued until last follow-up (median follow-up was 41 months) in 76.5% of patients. Löwenberg et al. [25] published a randomized placebo-controlled trial from the Netherlands for patients with active UC who were randomized for TDM-guided MP treatment. In their study, the primary endpoint was corticosteroid-free clinical remission and endoscopic improvement (total Mayo score ≤ 2 points and no item > 1) at week 52. In this study, it was allowed for patients who were “shunters” to add allopurinol; TG was not allowed in this study. Addition of allopurinol was required in 14 out of 29 (48.3%) patients. This primary endpoint was achieved in 48.3% of patients on MP and in only 10% using placebo (*p* = 0.002). Adverse events occurred more frequently with MP (808.8 per 100 patient-years) compared to placebo (501.4 per 100 patient-years). Unfortunately, despite being an efficacious treatment for CD and UC, up to 50% of patients cease thiopurine treatment within the first two years due to intolerance or ineffectiveness [26]. Therefore, innovating dosing for thiopurines should be introduced in daily practice such as low-dose classical thiopurines in combination with allopurinol (AzaAllo) or low-dose thioguanine (TG) therapy to improve efficacy and prevent intolerance [18]. In the following sections, the different innovative thiopurine treatment strategies will be discussed.

## Low-dose azathioprine/allopurinol combination therapy

AZA/allopurinol therapy (AzaAllo), 25–50 mg/100 mg, is a properly reported approach for IBD, firstly reported in 2005 for its use in IBD by Sparrow et al. [27]. This innovative approach was primarily used for “shunting” patients who had relative high amounts of 6-methyl mercaptopurine ribonucleotides (6-MMP) in ratio to 6-thioguanine nucleotides (6-TGN) and are thus more susceptible to hepatotoxicity induced by high 6-MMP and reduced efficacy from low 6-TGN concentrations. When combined with low-dose azathioprine dosed in 25–50 mg (25% to 33% of regular dose), 100 mg of allopurinol enhances the conversion of azathioprine into its active metabolites, 6-TGN, by inhibiting the enzymes responsible for the breakdown of 6-MP into 6-MMP and 6-thiouric acid. This leads to higher intracellular 6-TGN levels, responsible for the therapeutic effects of AZA [28]. The risk of hepatotoxity (and myelotoxicity) is dramatically reduced, as high 6-MMP is associated with developing hepatotoxicity, not only in the first months of therapy, but sometimes even after 10-15 years [29, 30]. Regarding the usefulness of AzaAllo, various retrospective studies have reported long-term efficacy and safety [31–36]. A randomized-controlled trial was recently reported (2020) in UC patients in Denmark who were steroid-dependent/refractory, thiopurine naïve, had a normal thiopurine methyltransferase and achieved remission with steroids or infliximab. They were randomized to either AzaAllo or AZA monotherapy. It was found that after 52 weeks, 43% patients in the AzaAllo group and 21% patients in the AZA group achieved remission (odds ratio 2·54 [95% CI 1·00 to 6.78, *p* < 0·048]). Fourteen patients (30%) in the AzaAllo group and 16 (38%) in the AZA group were withdrawn from the study due to adverse events. Recently, Vasudevan et al. [37] published the results of the multicenter, randomized placebo-controlled DECIDER trial, which evaluated the low-dose thiopurine-allopurinol combination (LDTA) compared to standard thiopurine therapy plus placebo in IBD patients. In this study, the primary outcome was the proportion of patients achieving remission (Harvey Bradshaw Index < 5 for Crohn’s disease, Simple Clinical Colitis Activity Index < 4 for ulcerative colitis) and a fecal calprotectin < 150 μg/g after 26 weeks of treatment. The primary outcome was achieved in 50% of patients in the LDTA group, compared to 35% in the standard thiopurine group (*p* = 0.14). Furthermore, fewer participants stopped their allocated therapy due to adverse events (11% vs. 29%, *p* = 0.02) in favor of the LDTA group. Thiopurine dose adjustments were less frequently performed in LDTA group (69% vs. 92%, *p* = 0.03), which suggests optimal dosing is more easier achieved with LDTA. Furthermore, with regard to TDM, similar 6-TGN levels were obtained between LDTA and standard thiopurine groups (318 pmol/8 × 10^8^ RBC [IQR 213–456] and 312 pmol/8 × 10^8^ RBC [IQR 194–440], *p* = 0.77). More importantly, despite similar therapeutic 6-TGN levels, significantly lower 6-MMP levels were obtained in the LDTA group compared to the standard thiopurine group (139 pmol/8 × 10^8^ RBC [IQR 83–254] vs. 725 pmol/8 × 10^8^ RBC [IQR 140–2172], *p* = 0.005). Furthermore, liver function derangement occurred much less in the LDTA group compared to the standard thiopurine group (*n* = 2 [4%] vs. *n* = 11 [23%], *p* = 0.006). This suggests that indeed LDTA provides a biochemical advantage (i.e. lower 6-MMP values) compared to conventional thiopurines and that it is associated with significantly reduced abnormal liver function tests. However, it must be noted that the trial was terminated early due to slow recruitment. Based on these studies, the use of AzaAllo offers several important advantages. Firstly, it allows for reduced dosage of azathioprine, decreasing adverse effects while maintaining therapeutic efficacy. Secondly, the combination therapy optimizes drug metabolism and increases the proportion of azathioprine converted to its active metabolites. Lastly, it provides a viable affordable alternative for patients who are steroid-dependent/refractory or have previously failed and/or experienced intolerance to standard-dose azathioprine monotherapy and/or biological treatments. IBD specialists should discuss and consider AzaAllo as first-line treatment in patients with normal TPMT or NUD15, which might reduce the risk of side effects and the need to measure metabolites. In case of the unavailability of therapeutic drug monitoring (i.e. 6-TGN measurements), TG seems a better alternative than AzaAllo because 6-TGN levels might become too high after switching from AZA to AzaAllo. We advise to proactively measure 6-TGNs after switching to AzaAllo.

## Low-dose 6-thioguanine monotherapy

TG (0.2–0.3 mg/kg) is an emerging approach since 2001 in the treatment of IBD, particularly for patients who have not responded to or cannot tolerate conventional thiopurine therapies [38]. TG is currently only available on-label since 2022 in the Netherlands (Thiosix^®^ 10–20 mg) and off-label (Lanvis^®^ 40 mg) elsewhere, but offers an alternative option for IBD patients. It provides a potential avenue for achieving maintenance for IBD patients [14]. One of the major advantages of TG (0.2–0.3 mg/kg/day dosing) is that it is directly converted to 6-TGN without the formation of non-6-TGN metabolites that are formed in the conversion of MP (1.0–1.5 mg/kg) and AZA (1.0–3.0 mg/kg). While low-dose TG therapy holds promise, it is essential to consider its side effects. Like other thiopurines, TG may cause myelotoxicity, liver toxicity and gastrointestinal symptoms, but these occur at much lower frequencies compared to conventional thiopurines. Close monitoring of patients’ complete blood counts, liver function and adherence to therapy is necessary to ensure safety and optimal treatment outcomes [39]. Regarding the safety and efficacy of TG in IBD, a recent systematic review and meta-analysis from an Indian research group was reported [40]. They published that in 31 included studies; the pooled clinical response rate of TG therapy in IBD was 0.66 (95% C.I. 0.62–0.70; *I*2 = 16%). The duration of follow-up of the individual studies varied from two to 156 months. The pooled clinical response rate with low dose (≤ 20 mg/day) was similar to high dose (> 20 mg/day) TG therapy (0.65 [95% C.I. 0.59–0.70; *I*^2^ = 24%] and 0.68 [95% C.I. 0.61–0.75; *I*^2^ = 18%], respectively). Furthermore, the pooled remission maintenance rate was 0.74 (95% C.I. 0.61–0.84; *I*^2^ = 79%). The pooled rates of occurrence of nodular regenerative hyperplasia, liver function test abnormalities and cytopenia were 0.05 (95% C.I. 0.03–0.08; *I*^2^ = 75%), 0.12 (95% C.I. 0.08–0.16; *I*^2^ = 71%) and 0.06 (95% C.I. 0.04–0.09; *I*^2^ = 63%), respectively. The authors concluded that TG is a well-tolerated and efficacious therapy for maintenance in IBD. TG may also be considered first-line therapy in IBD [41].

## Combination therapy of biologicals with TG/AzaAllo

Combining the above-mentioned innovative strategies alongside biological therapies should be practiced when they become more available and affordable for the underprivileged patients. Use of biologics in the IBD-ENC cohort was only 4% for ulcerative colitis and 13% for Crohn’s disease [9]. As shown in Table 3, the most affordable medications available to patients are methotrexate injections ($42), thiopurines ($70) and mesalamine ($183) suppositories in India. These are the estimated annual costs for an IBD patient in India. The average monthly income in India is approximately $410 [42] and this might even be lower in IBD patients whose income is reduced by their inability to work due to their disease. This average income might even be lower in other countries in South-East Asia [20]. In comparison, the annual cost for biological therapies in India such as adalimumab ($2908), infliximab ($5270) and vedolizumab ($7520) is not within the purchasing power of the average Indian patient compared to methotrexate or thiopurines [20]. Thus, currently in LMICs, but unfortunately also in financially non-insured patients in high-income countries such as in the US, biological use is not common in the management of IBD if indicated due to high costs for such patients [18].

## Safe use of thiopurines

**Fig. 1:**
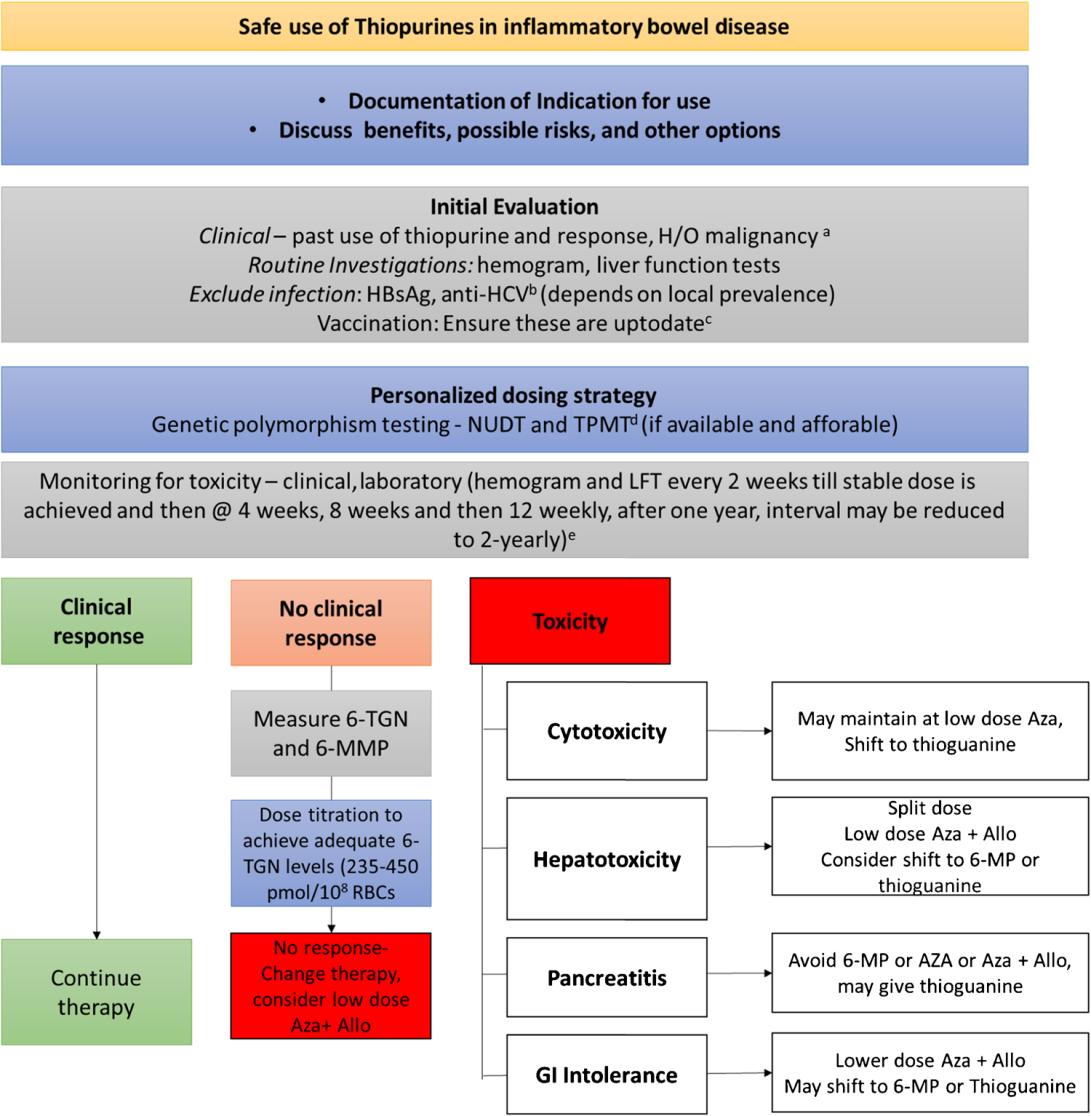
Schematic overview of the management of thiopurine-treated inflammatory bowel disease (IBD) patients. **a** Clinical history should determine individuals who may be at increased risk of adverse events like those with previous pancreatitis, liver disease, and cytopenias, or those who had previous alcohol abuse. **b** Many experts recommend additional tests such as Varicella Zoster virus (VZV) and Epstein-Barr virus (EBV) serology, but the cost-effectiveness of these tests is unclear.. ^c^Vaccinations, as recommended for IBD patients, should be considered and administered. Vaccinations depends on local prevalence. ^d^Genotype testing can guide initial thiopurine dosing. The choice of genotype testing should be based on geographic predominance (e.g. nucleotide diphosphatase [NUDT] in India and Asia). ^e^While the frequency of monitoring may be reduced with time, it should never be stopped as cytopenias could occur at any time. We typically target a total leukocyte count of 4000/mm^3^, although a leukocyte count till 3000/mm^3^ may be safe

Since thiopurines have been used for a considerable period of time, there is extensive IBD literature about the adverse effects and the optimal use of these drugs [17]. Classical thiopurines, as previously mentioned, are useful as a maintenance therapy. The indications for starting thiopurines can be found in Table 1. Therefore, it is of extreme importance to start these in a timely manner so that the maximal action is achieved by the time the induction therapy (typically steroids) is tapered. Thiopurines should not be started during active bacterial, mycobacterial, viral or fungal infection. It is important to be up to date with vaccinations as use of immunomodulating therapies is well recognized to pre-dispose to infections (Fig. 1). The vaccination recommendations differ by country and region. It would be advisable to follow-up local guidelines regarding vaccinations. There is now consistent evidence that the recognition of certain genetic polymorphisms in TPMT and NUDT15 could identify the subset of patients at an increased risk of thiopurine-related cytopenia. Similar to other Asian countries, evidence from South Asia supports the use of NUDT15 testing [43–45]. Where affordable and applicable, these should be considered prior to starting thiopurines in (migrant) Asian populations. In homozygous mutants, the use of thiopurines should be avoided (or use very low dose 0% to 10%), while in heterozygotes, 25% to 50% of the standard dose should be given. One should be aware of potential drug interactions of thiopurines; for example, allopurinol inhibits xanthine oxidase and increases thiopurine metabolites. One should be aware of other adverse effects such as pancreatitis, hepatotoxicity, flu-like syndrome and gastrointestinal tolerance. Most of these are believed to be idiosyncratic (Table 2). Thiopurine-related hepatotoxicity is believed to be largely mediated through 6-MMP, which may be formed in increased amounts due to shunting [46]. Furthermore, TDM strategies may monitor thiopurine therapy by using 6-TGN [47]. This strategy can be quite costly and as an alternative monitoring mean corpuscular volume (MCV) may be used [48]. MCV levels rise during thiopurine therapy and can be used as a surrogate marker for thiopurine therapy compliance, in settings where 6-TGN measurements are not available [48]. Thiopurines can cause both an elevation of aminotransferases and thrombocytopenia. Chronic liver injury may result in veno-occlusive disease or nodular regenerative hyperplasia (NRH). While mild aminotransferase elevations may disappear with continuation of therapy, clinically manifest toxicity warrants drug cessation. In such situations, re-challenge should be avoided.

**Table 1:** Indications of thiopurines in inflammatory bowel disease. *IFX* infliximab *CD* Crohn's disease, *UC* ulcerative colitis

	Ulcerative colitis	Crohn’s disease
Clear indications	• Steroid-dependent UC • Two courses of steroids in 1 year • Acute severe colitis where remission was induced with cyclosporine • In combination with biologicals (IFX)	• Inflammatory phenotype where remission was achieved with steroids • In combination with biologicals (anti-TNF) • Postoperative CD
Less established indications	First episode of Acute Severe Ulcerative Colitis (ASUC) together with IFX [49]	Fistulizing CD [15, 50] (only in combination with IFX) Stricturing CD [51] (only in combination with IFX)

**Table 2:** Overview of recommendations that can be considered in patients starting AzaAllo or 6-TG therapy. These guidelines and positions are intended to support daily practice. This guideline is based on the medication guideline IBD (allopurinol in combination with azathioprine/mercaptopurine) of the Dutch Gastroenterology Society [52]

Brand names	Mercaptopurine (Puri-Nethol^®)^ Tablet 50 mg Azathioprine (Imuran^®^) Tablet 25 mg, 50 mg Allopurinol Tablet 100 mg Thioguanine (Thiosix^®^, Lanvis^®^) Tablet 20 mg, 40 mg
Dosages	**AZA monotherapy** 1.0–3.0 mg/kg/day **MP monotherapy** 1.0–1.5 mg/kg/day **Thiopurine-allopurinol combination:** Mercaptopurine 1 od 0.25–0.375 mg/kg/day usually 25 mg increase dose based on 6-TGN level Azathioprine 1 od 0.5–0.70 mg/kg/day usually 50 mg, increase dose based on 6-TGN level Allopurinol 1 od 100 mg/day **Thioguanine monotherapy** Thioguanine 1 od (0.2–0.3 mg/kg/day) usually 20 mg (up to 40 mg/day), no dose adjustments necessary based on 6-TGN level
Therapeutic effect	2–3 months
Interactions	• 5-ASA: higher 6-TGN levels/bone marrow depression [53] • Allopurinol: higher 6-TGN levels (active metabolite) and lower 6-MMP levels (side-metabolite). [28] • Ribavirine: (increased likelihood of bone marrow depression) [54]
Fertility	• Fertility: no adverse effects, based on current literature [55] • Pregnancy: sparse data available. Caution is advised [56, 57] • Lactation: Allopurinol passes into breast milk. Consequences not clear. Be very cautious with the use of allopurinol. [58, 59]
Lab prior to therapy	• Hb, MCV, leukocytes + differentiation, trombocytes • Kreatinine + eGFR • ASAT, ALAT, alkaline phosphatase, gamma-GT, bilirubin (total) • In case of low Hb: ferritin and transferrin saturation • Hepatitis B and C screening and EBV status • On indication: CMV • Feces calprotectine and/or endoscopy
Lab during therapy	Weeks 1. 2, 4, 6, 8, and 12: Hb, MCV [48] (might be increased during thiopurine usage), trombocytes, leukocytes + differentiation, kreatinine, alkaline phosphatase, bilirubin (total), gamma-GT, ASAT, ALAT Afterwards, every three months during first year: Hb, MCV, trombocytes, leukocytes + differentiation, kreatinine, alkaline phosphatase, bilirubin (total), gamma-GT, ASAT, ALAT After first year of thiopurine usage, every six months: Hb, MCV, trombocytes, leukocytes + differentiation, kreatinine, alkaline phosphatase, bilirubin (total), gamma-GT, ASAT, ALAT
Drug levels	**AZA/6-MP** Therapeutic levels 6-TGN: 235–450 pmol/8^*^10^8^ RBC (Lennard Method) 6-TGN: 300–600 pmol/8^*^10^8^ RBC (Dervieux Method) Toxic levels 6-MMP: > 5700 pmol/8^*^10^8^ RBC In case of low 6-TGN and high 6-MMP: 25% of current dosage and add 100 mg allopurinol under frequent follow-up of general lab and 6-TGN levels **6-TG** Therapeutic levels 6-TGN: 235–1000 pmol/8^*^10^8^ RBC (Lennard Methode) 6-TGN: 300–1250 pmol/8^*^10^8^ RBC (Dervieux Methode) Toxic levels 6-TGN: > 2000 pmol/8^*^10^8^ RBC (Lennard Method) 6-TGN: > 2600 pmol/8^*^10^8^ RBC (Dervieux Method)
TPMT	**AZA/6-MP** * TPMT intermediate metabolizer* Start with 50% of the standard dose [60] * TPMT poor metabolizer* 1. 10% of standard dosage [60] 2. Choose other alternative * Thiopurine-allopurinol combination* Only use in TPMT normal metabolizer [61] **Thioguanine therapy** * TPMT intermediate metabolizer* Start with 50% of the standard dose (0.1–0.15 mg/kg/day) [60] *TPMT poor metabolizer* 1. TG 20 mg every week [62] 2. Choose other alternative * It must be noted that the evidence of the use of genotyping in TG therapy is not clear
NUDT15	**AZA/6-MP** * NUDT15 intermediate metabolizer* Start with 25% to 50% of the standard dose * NUDT15 poor metabolizer* 1. Avoid thiopurines, or 2. If cannot be avoided use 10% of standard dosage **Thiopurine-allopurinol combination** Only use in NUDT15 normal metabolizer **Thioguanine therapy** *NUDT15 intermediate metabolizer* Start with 25% to 50% of the standard dose *NUDT15 poor metabolizer* 1. Avoid TG, Or if cannot be avoided, 2. Use 25% to 50% of standard dose TG every week

*AZA* Azathioprine, *MCV* mean corpuscular volume, *MP* mercaptopurine, *NUDT15* nudix hydrolase 15,  *TDM *therapeutic drug monitoring, *TG* thioguanine, *TPMT* thiopurine S-methyltransferase, *6-MMP* 6-methyl mercaptopurine ribonucleotides, *6-TGN* 6-thioguanine nucleotides

## Additional safety issues of thiopurines

### Nodular regenerative hyperplasia

NRH is a rare liver condition characterized by the development of small regenerative nodules throughout the liver [63–65]. It has been reported in association with the use of thiopurines, particularly azathioprine, although the overall risk is relatively low. The exact incidence of (symptomatic) NRH in individuals taking thiopurines is not well established and data on its prevalence in this specific population are limited. However, a study by van Asseldonk et al. [66] suggested that the risk of developing (symptomatic) NRH while on low-dose TG therapy in IBD is 6%; interestingly, the background incidence of NRH within IBD is also around 6% [67, 68]. In non-IBD patients, the incidence is around 2% [69]. NRH is generally considered a dose-related phenomenon; it is important to note that a vast majority of individuals who take thiopurines do not develop NRH. Toksvang et al. [68] wrote a comprehensive, systematic review covering the risk of developing NRH in TG-treated patients. For TG, it was found that there is a dose-related relationship, as most cases who developed NRH were using higher dosages (> 20 mg/day) of TG [70]. The authors stated that dosages at or below 12 mg/m^2^/day are rarely associated with notable hepatotoxicity and TG can probably be considered safe [68].

### Risk of malignancy

In the past, there have been concerns that thiopurines might increase the risk of cancer, particularly lymphoma, leukemia and skin cancer. The increased risk of malignancy with thiopurines is thought to be related to immunosuppression and subsequent viral infection (e.g. EBV infections) [71] or deoxyribonucleic acid (DNA) damage (higher susceptibility of DNA-TG to oxidation) [72]. In a Dutch study, thiopurine use (AZA/MP) was found in 92% of EBV-positive lymphomas, while in EBV-negative lymphomas only 19% of patients used thiopurines. This suggests that a correlation may exist between the use of thiopurines and the risk of developing EBV-positive lymphoma. Furthermore, a study found that cultured human cells incorporated with TG in the DNA (DNA-TG) who were exposed to ultraviolet A produced more DNA-TG oxidation products that inhibit transcript elongation by blocking RNA-polymerase II. This suggests that thiopurines may have carcinogenic hazard risk. But, conflicting results have been published in the literature regarding the thiopurine-related incidence of cancer [73–76]. The discrepancies might reflect different ranges of treatment duration, patient age and disease severity and the risks of long-term treatment might have been underestimated [72]. Lemaitre et al. [65] performed a nationwide cohort study in France on the risk of lymphoma in IBD patients. In this cohort study of 189,289 patients with IBD, the risk of incident lymphoma was significantly higher in patients exposed to thiopurine monotherapy (adjusted hazard ratio 2.60; 95% CI, 1.96–3.44; *p* < 0.001), anti-TNF monotherapy (aHR, 2.41; 95% CI, 1.60–3.64; *p* < 0.001) or combination therapy (aHR, 6.11; 95% CI, 3.46–10.8; *p* < 0.001) compared with those who were unexposed. The risk was higher in patients exposed to combination therapy vs those exposed to thiopurine monotherapy (aHR, 2.35; 95% CI, 1.31–4.22; *p* < 0.001) or anti-TNF monotherapy (aHR, 2.53; 95% CI, 1.35–4.77; *p* < 0.001). The authors concluded that the use of thiopurine monotherapy or anti-TNF monotherapy was associated with a small but statistically significant increased risk of lymphoma and that this risk was higher with combination therapy. A study performed by Ranjan et al. [77] found that the risk of developing lymphoma was minimal in IBD patients using AZA in northern India. In 1093 IBD patients (UC = 72%) who received thiopurines for over three months (of whom 23.2% received thiopurines for more than five years); no patients developed lymphoma or non-melanoma skin cancer. A study from Japan also did not find evidence of increased risk of non-Hodgkin lymphoma in thiopurine-treated patients, but did find evidence that thiopurines may cause non-melanoma skin cancer [78]. However, it is important to note that the absolute risk of developing malignancy while taking thiopurines is generally considered to be low. Otherwise, there are reports that TG might inhibit the development of colitis-associated colon cancer [79–81]. The benefits of thiopurine therapy in managing the underlying disease outweigh the potential risks. More research is needed to fully establish the role of thiopurines on the development of various types of cancer such as lymphoma and non-melanoma skin cancer.

## How to highlight innovating thiopurines among IBD community and patients

Currently, there is a lack of prospective studies being performed using thiopurines for IBD due to a lack of industrial funding because thiopurines are generic drugs [11, 14]. As highlighted in this article, thiopurines still have a major role in the management of IBD, not only because of the affordability of these drugs. Many IBD patients in developing countries can only afford thiopurines, while biologicals and newer small molecule therapies are not yet financially available to these patients. However, most of the current research within the IBD landscape is performed for biologicals and newer small molecules (e.g. Janus kinase [JAK] inhibitors). Therefore, patients in LMICs are double burdened because they cannot access expensive therapies, if indicated and there is a lack of ongoing research on these drugs (e.g. thiopurines/methotrexate) that are used for their IBD. Gastroenterologists in LMICs do perform studies regarding local IBD patients using thiopurines, contributing extensively to the body of scientific data on the effectiveness and safety of thiopurines [17, 21, 40, 77, 82, 83]. However, in our opinion, more funding should be directed to innovating thiopurine evaluations in order to continue to improve current therapies for the better good, especially considering that patients in LMICs and underprivileged unassured patients in high-income countries use classical thiopurines as IBD maintenance therapy as their only option. Traditionally, classical thiopurines have been used in weight-based doses. This results in toxicity in about 30% to 50% of patients needing withdrawal of the drug [24]. Pre-emptive testing for TPMT variants identifies in about 10% of these patients [84]. NUD15 variants like rs116855232 might be linked to thiopurine toxicity [85]. In high-risk populations, this genotyping can be considered. If not available, simple liver and blood count testing should be done, in case of side effects and/or inefficacy. AzaAllo or TG should be discussed and applied before moving to early biological therapies (Table 3).

**Table 3:** Annual costs of pharmacological therapies for IBD in India. Cost of medication has been calculated for an average adult weighing approximately 60 kg. The average monthly income in India is estimated to be approximately US$411. Based on the figures by Balasubramaniam et al. [20]

Drug	Costs ($)
Prednisolone	2 (per induction course)
Methotrexate injection	42
Thiopurines	70
Hydrocortisone enema	160 (1 g for a year)
Mesalamine supppository	183
Oral mesalazine	604
Mesalamine foam	997
Tofacitinib generics	368
Adalimumab biosimilar	2.908
Infliximab biosimilar	4.512
Infliximab	5.270
Vedolizumab	7.520
Tofacitinib	11.580

Thiopurines are cost-effective and affordable, which benefits patients in LMICs. Strategies such as AzaAllo and TG can be used not only in patients who previously failed and/or experienced intolerance to standard-dose thiopurines, but even as first-line therapies to improve the chance of treatment success and reduce risk of toxicity at an early stage of treatment. However, so far we do not know which therapy strategy (AzaAllo or TG) is the most optimal as first-line therapy and warrants more research. TG seems preferable to AzaAllo in (suspected) non-compliant patients, as only one pill needs to be given daily and therapy non-adherence has been shown to increase with increase in the number of pills per day. Also, TG seems safer in cases of pregnancy, because allopurinol cannot be given safely then and there is increasing evidence for safe use in pregnancy of thioguanine [56]. A majority of the AZA-related side effects occur within the first six months of starting AZA with less than 2% of patients developing a new onset of side effects beyond five years of AZA usage [24]. This underlines the need for optimal outpatient clinic control in the first six months after starting thiopurines. Long-term AZA therapy directed by thiopurine metabolite levels and metabolic enzyme activity appears to be reasonably safe and clinically effective for maintaining durable clinical remission in IBD. Evaluating AzaAllo and/or TG is urgently needed for the 30% to 50% of patients who cannot tolerate the classical thiopurines, to keep IBD patients on innovated, affordable and available thiopurine treatment before switching early to biological treatments.

In conclusion,  the prevalence of IBD is on the rise not only among the rich, but also in underprivileged patients and one of the efforts to address this growing burden should be to focus on an innovative and inclusive thiopurine approach to mitigate the impact of IBD on individuals and healthcare systems in LMICs. Improving of proper dosing of thiopurines feels like an uphill battle. In case of inefficacy or side effects, we would prefer TG over AzaAllo, because supposedly AzaAllo might still produce some side metabolites such as 6-MMP, while TG is directly metabolized to 6-TGNs. Moreover, dosing two prescriptions instead of one also influences the compliance of those drugs.

## Data Availability

Not applicable.

## Declarations

### Disclaimer

The authors are solely responsible for the data and the contents of the paper. In no way, the Honorary Editor-in-Chief, Editorial Board Members, the Indian Society of Gastroenterology or the printer/publishers are responsible for the results/findings and content of this article.
